# Nonlocal accumulation, chemical potential, and Hall effect of skyrmions in Pt/Co/Ir heterostructure

**DOI:** 10.1038/s41598-020-57818-w

**Published:** 2020-01-23

**Authors:** Satoshi Sugimoto, Wataru Koshibae, Shinya Kasai, Naoki Ogawa, Yukiko Takahashi, Naoto Nagaosa, Yoshinori Tokura

**Affiliations:** 1 Research Center for Magnetic and Spintronic Materials, National Institute for Materials Science (NIMS), 1-2-1 Sengen, Tsukuba, 305-0047 Japan; 2 RIKEN Center for Emergent Matter Science (CEMS), Wako, 351-0198 Japan; 3 JST, PRESTO, 4 - 1 - 8 Honcho, Kawaguchi, Saitama 332 - 0012 Japan; 4 Department of Applied Physics, University of Tokyo, Tokyo, 113-8656 Japan; 5 Tokyo College, University of Tokyo, Tokyo, 113-8656 Japan

**Keywords:** Spintronics, Magnetic devices

## Abstract

Magnetic skyrmion is a swirling topological spin texture behaving as an individual particle. It shows a gyro-motion similarly to that of a charged particle under a magnetic field, being led to the transverse shift to the electric current, i.e., skyrmion Hall effect. With the open boundaries of a sample, this results in an accumulation of skyrmions on one side and their depletion on the other side. Here we demonstrate experimentally that this effect propagates non-locally over tens of micrometers even where the electric current is absent, when the narrow wires bridge bar-shaped Pt/Co/Ir heterostructure thin film systems. This nonlocality can be understood in terms of the “chemical potential” gradient for the skyrmion bubble induced by the skyrmion Hall effect in the nonequilibrium steady state under the electric current. The present result shows that the skyrmion Hall effect acts as the skyrmion pump and the thermodynamic concepts can be applied to the aggregate of skyrmion bubbles.

## Introduction

Magnetic skyrmion^1–6^ is a two-dimensional topological spin texture characterized by an integer called skyrmion number^7^ which counts how many times the spin directions wrap the unit sphere. Skyrmions have been firstly observed in the form of the triangular lattice array, i.e., skyrmion crystal (SkX), in bulk crystals of B20 compounds, e.g., MnSi^8–10^, Fe-Co-Si^11^, and FeGe^12–14^. This SkX may be regarded as the superposition of the spin density waves of three wavevectors, i.e., magnetically two-dimensional systems^9,15,16^. In addition, it has been recently shown that the gas phase of skyrmions exists near the boundary of SkX^17,18^, where the individual skyrmion behaves as a stable particle protected by the topology. In this gas phase, the picture of the close-packed repulsively interacting skyrmions is more appropriate rather than the superposition of three spin density waves of SkX. This suggests the possible transfer and accumulation of skyrmions by external means in sharp contrast to the density waves or solids where the lattice constant cannot be changed so much^19,20^.

From the viewpoint of applications, skyrmions at room temperature are highly desired, as realized in *β*-type Co-Zn-Mn alloys^21^. Another strategy to develop the room-temperature skyrmions has been established in artificial thin-film heterostructures using spintronics-friendly materials^22–31^. In some of these systems, sub-*μ*m scale skyrmions, called as skyrmion bubbles where the dipolar contribution is dominant, can be stabilized locally with breaking triangular lattice symmetry of SkX. Such local stabilizations are attributed to the film inhomogeneity^23^ and/or the design the structures^22^, and provide the systems to study individual transfers and accumulation behaviors as the picture of the gas-like phase experimentally.

In the present study, using Pt/Co/Ir heterostructured thin-film^24,32–34^ samples, we study the current-induced motions of skyrmion bubbles and their mass current at the steady state. The topological nature of the skyrmion bubbles appears in the electric response: Because of the vorticity of the characteristic winding magnetic texture, the Magnus effect^35^, i.e., the perpendicular response to the current, occurs. A typical outcome of this effect is the Hall motions of the current-driven skyrmions^36–40^. The impurity scattering also causes similar behavior during the current-induced motions^41–46^. Such transverse motions of skyrmion bubbles will cause the skyrmion accumulation along the open end of the sample perpendicular to the current direction^39,47^. In a steady state of the sample with the open ends where skyrmion creation/annihilation does not occur, the mass skyrmion current perpendicular to the skyrmion flow is zero. This open-end condition perpendicular to the current direction naturally leads us to the spatially inhomogeneous distribution of skyrmion bubbles, which can be described by “skyrmion chemical potential”. By the application of this thermodynamic concept, we propose a guiding principle to control the aggregate of skyrmion bubbles.

**Figure 1:**
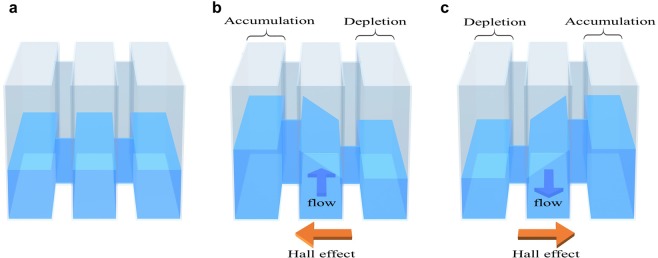
Schematic images for accumulation, chemical potential and skyrmion Hall effect, where the liquid represents the skyrmion aggregates. Three parallel canals are connected by narrow gate channels through which liquid can flow. The canals are filled by a liquid which shows a Hall effect. (**a)** The system without flow. The heights of the surface are horizontally identical. (**b**) Only on the middle canal has a flow. Due to the Hall effect, a directional accumulation/depletion perpendicular to the flow occurs. Therefore, at the steady flow on the middle canal maintains a difference in the surface heights between left and right canals. (**c**) The same as (**b**) but direction of flow on the middle canal is opposite.

Figure 1 shows schematic images to illustrate the accumulation, chemical potential and Hall effect of skyrmions: three canals are filled by liquid which shows a Hall effect. The canals are connected by very narrow gate channels through which liquid can flow. Therefore, the height of the surface, i.e., chemical potential, is equal through the gate channels. In the system without flow, i.e., at the ground state, the surface height of the liquid is equivalent over the whole system. When the liquid in the middle canal flows in a steady state, the Hall effect induces the gradient of the surface in the perpendicular direction to the flow. As a result, a difference in the surface height of the fluid between the left and right canal is achieved and maintained by the fluid flow in the middle canal.

**Figure 2:**
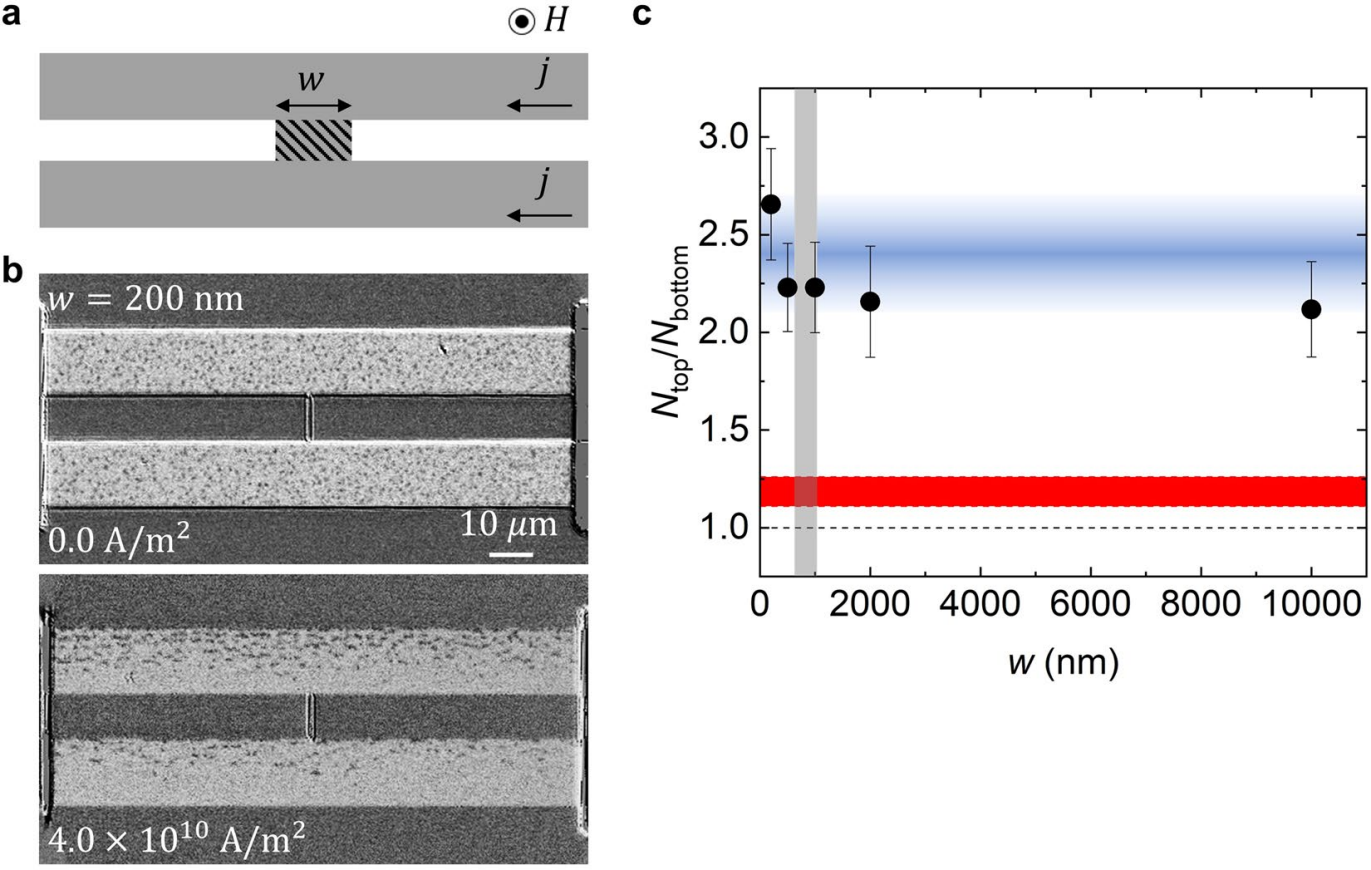
Design rule of the skyrmion gate channel. (**a**) The schematic representation of the device setup. (See Methods and ref. ^47^). Two skyrmion-conducting bars (gray areas) are bridged by the junction with a width *w* (hatched area). The same current density *j* is applied to the top and the bottom bars, so that the electric current through the junction does not flow. The arrow under the character *j* indicates the positive direction of the electric current. (**b**) The p-MOKE images for *j* = 0 (top) and *j* = + 4.0 × 10^10^ A/m^2^ (bottom), under the condition with {*H* = +3.8 Oe, *w* = 200 nm} and 10 μm for the length of the junction. The skyrmion bubbles are observed as the dark spots. (**c**) The junction width *w* dependence of the ratio *N*_top_/*N*_bottom_ where *N*_top_ (*N*_bottom_) is the number of skyrmion bubbles on the top (bottom) bar for *j* = +4.0 × 10^10^ A/m^2^. We confirm that in the system without current injection, i.e., for *j* = 0, *N*_top_/*N*_bottom_ ≈ 1. Without the gate channel, *N*_top_/*N*_bottom_ shows a small deviation from 1 due to the Oersted field effect of the electric current *j* (the red region). With the gate channel, *N*_top_/*N*_bottom_ is almost independent of the width *w*, showing *N*_top_/*N*_bottom_ ≈ 2.3 as guided by a blue band. Note that the skyrmion accumulation/depletion occurs even in the case that *w* is smaller the skyrmion bubble size indicated by the gray region.

**Figure 3:**
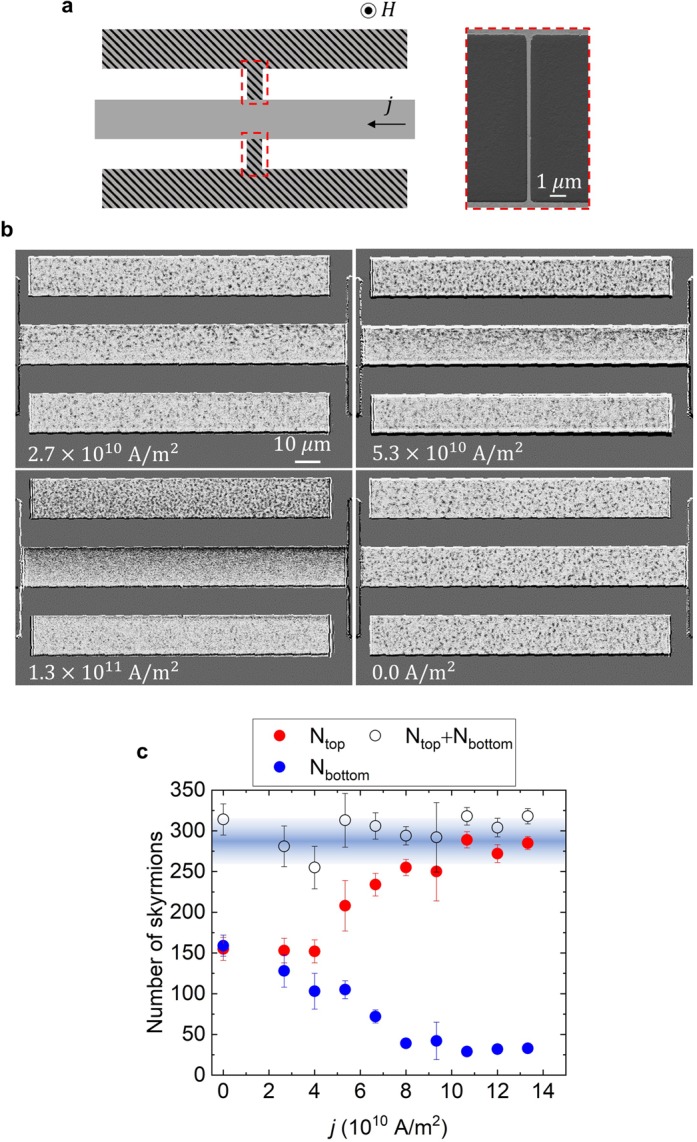
Skyrmion pumping by local current injection. (**a**) The device structure. The left figure is a schematic representation. Three skyrmion-conducting bars (gray areas) are bridged by two junctions with a width 200 nm (red frames). The current is locally injected in the gray area only, and electric current is *not* applied at the hatched areas. The arrow in the gray region indicates the positive direction of the current *j*. The right picture is the SEM image of the junction. (**b**) The p-MOKE images for *j* = +2.7 × 10^10^ A/m^2^, +5.3 × 10^10^ A/m^2^, +1.3 × 10^11^ A/m^2^ with *H* = +3.8 Oe are shown. The junction width *w* and its length *l* are set to be *w* = 200 nm and *l* = 10 μm, respectively. After those current applications, the current is tuned off. The relaxed state p-MOKE image (*j* = 0) is presented at right bottom of the panel (**b**). This skyrmion spatial distribution is nearly identical with the *j* = 0 initial state. The junction width (*w* = 200 nm) is too narrow to be detected by the p-MOKE observation used here. (See text and Methods). (**c**) The number of skyrmion bubbles *N*_top_ (*N*_bottom_) on the top (bottom) bar is plotted by red (blue) circles with error bar as a function of *j*. The net number of skyrmion bubbles on the top and bottom bars *N*_top_ + *N*_bottom_ is also presented by open circles. This net number barely depends on *j*, and stays almost constant *N*_top_ + *N*_bottom_ ≈ 300 as eye-guided by a blue band.

**Figure 4:**
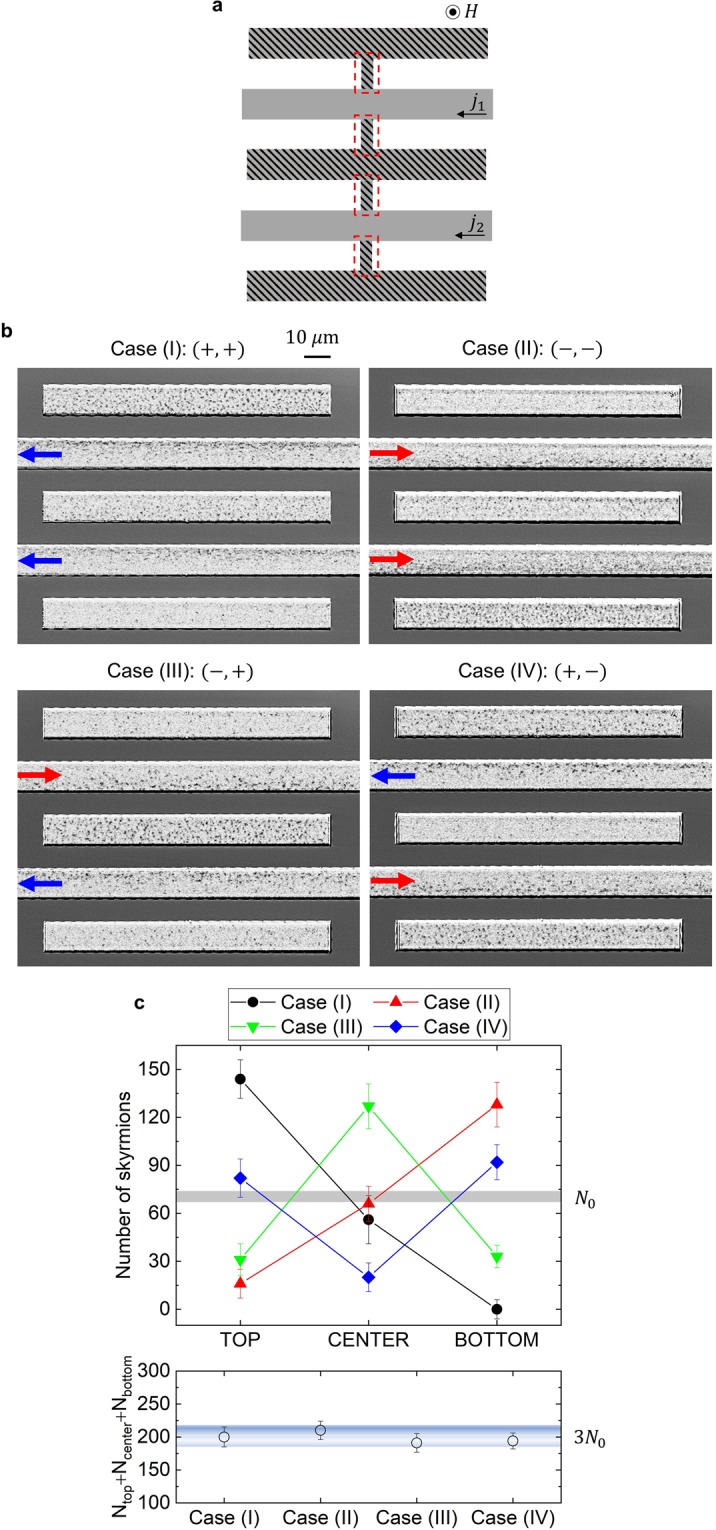
Skyrmion pumping in 5-bar system. (**a**) Schematic representation of the device structure. Five skyrmion-conducting bars (gray areas) are bridged by four junctions with a width 200 nm same with the setup in Fig. 3 (red frames). The currents *j*_1_ and *j*_2_ are locally injected in the two gray areas only, and electric current does *not* flow at the hatched areas. The arrow in the gray region indicates the positive direction of the current *j*_1_ and *j*_2_. (**b**) The p-MOKE images for four cases. The bridging junctions are invisible, similar to the results shown in Fig. 3b. Case (I): (+, +), *j*_1_ and *j*_2_ are both positive. Case (II): (−, −), *j*_1_ and *j*_2_ are both negative. Case (III): (−, +), *j*_1_ is negative and *j*_2_ is positive. Case (IV): (+, −), *j*_1_ is positive and *j*_2_ is negative. The images are observed under the condition with | *j*_1_ | = | *j*_2_ | = 5.0 × 10^10^ A/m^2^ and *H* = +2.7 Oe. (See also Supplementary Information 2). (**c)** Upper panel: The number of skyrmion bubbles on the top (*N*_top_), the center (*N*_center_) and the bottom (*N*_bottom_) bars for the cases (I)~(IV). The gray bar indicates the number in the equilibrium state (*N*_0_) without current injection (*j*_1_ = *j*_2_ = 0) with average error. Lower panel: The net number of skyrmion bubbles *N*_top_ + *N*_center_ + *N*_bottom_ for the cases (I)~(IV). It shows almost the same value for all the cases, approximately 3*N*_0_ as eye-guided by a blue band.

In accord with the case of the above consideration, the nonlocal accumulation and depletion of the skyrmions indicate the spatial profile of the skyrmion chemical potential. In other words, we can control and design the aggregate of skyrmions by local current. In the following, the design rule of such skyrmion devices is discussed with the experimental results summarized in Figs. 2 and 3. We then show the experimental evidence to support the idea of skyrmion chemical potential in Fig. 4.

## Results

### Design rule of the sufficiently narrow skyrmion gate channel

First, we study the response of the skyrmion aggregate to the current injection using two Pt/Co/Ir bars bridged by a narrow junction, modeled as the *skyrmion-conducting bars* and a *gate channel* after Fig. 1. Figure 2a shows the schematic representation of the device setup: two skyrmion-conducting bars are connected by the junction with width *w*, and electric current is injected to both bars. (For the details of the device preparation, see Methods and ref. ^47^). Here, skyrmion bubbles are considered to be mainly driven by the spin-orbit torque within conducting bars (the gray regions), and the electric current through the junction does not flow (the hatched region). In this experimental setup, we examine the spatial distribution of the skyrmion bubbles in the current induced nonequilibrium steady state.

Figure 2b shows the results of the real-space observations by the polar magneto-optical Kerr (p-MOKE) effect. The length and width of the junction are *l* = 10 μm and *w* = 200 nm, respectively. Here, the skyrmion bubbles are observed as randomly distributed dark spots as shown in the top panel of Fig. 2b. Similar to the previous study^47^, we see the skyrmion Hall response to the current injection; the skyrmion bubbles are gathering at the upper edge at each skyrmion-conducting bar as clearly seen in the bottom panel of Fig. 2b. Beyond this skyrmion Hall behavior, we find a large difference in the number of skyrmion bubbles between the top and the bottom bars.

Figure 2c shows the ratio *N*_top_*/N*_bottom_, where *N*_top_ (*N*_bottom_) is the number of skyrmion bubbles on the top (bottom) bar at the current-induced nonequilibrium steady state, as a function of the junction width *w* for *j* = + 4.0 × 10^10^ A/m^2^. (The definition of the sign of *j* is indicated in Fig. 2a. See also Methods for the estimation of the number of skyrmion bubbles.) We find that *N*_top_ is always more than twice as large as *N*_bottom_; the average of *N*_top_*/N*_bottom_ is about 2.3 and is almost independent of *w* over the range of 200 nm ⩽ *w* ⩽ 10 μm. This difference between *N*_top_ and *N*_bottom_ is found even in the case that *w* is smaller than the present skyrmion bubble size *d* (630 nm ⩽ *d* ⩽ 1040 nm: Supplementary Information 1) indicated by gray area in Fig. 2c.

Let us discuss the current-induced difference between *N*_top_ and *N*_bottom_. For a control experiment, we examine the case that *w* = 0, i.e., the top and the bottom bars are isolated. (See also Supplementary Information 2). In this case, we confirm *N*_top_*/N*_bottom_ ≈ 1 with a slightly positive deviation which is indicated by red region in Fig. 2c. This small deviation may come from the effect of the Oersted field by the electric current, i.e., in addition to the external magnetic field, the top (bottom) bar is affected by the Oersted field due to the electric current on the bottom (top) bar.

The experimental results summarized in Fig. 2 clearly show that the width *w* = 200 nm is enough to exert the junction effect, which induces significant difference between *N*_top_ and *N*_bottom_. This observation indicates that skyrmion bubbles can pass through the junction even in the case that the junction width is smaller than the minimum of the steady-state skyrmion bubble size *d* ∼ 630 nm. In the previous study^48^, the flexibility of the skyrmion has been demonstrated; transformation between the magnetic textures, e.g., a skyrmion and a domain wall pair, occurs during the motion of those magnetic textures passing through the narrow path. Although the experimental technique used in the present study is not able to resolve such fast transient evolution of the magnetic textures in the narrow junction, the experimental visualization of the dynamics will be interesting as a future challenge.

In addition to the design rule of the narrow gate channel, we also find a strategy to prepare the skyrmion-conducting bar arrangements. As discussed above, about 10 μm separation of the skyrmion bars is enough to reduce the Oersted field effect. On the basis of the knowledge summarized in Fig. 2, we can design the devices as shown in the following and discuss the skyrmion response to the current density *j* beyond the Oersted field effect.

### Skyrmion pumping beyond several tens of micrometers by skyrmion Hall effect

Next, let us examine the local current injection effect on the skyrmion accumulation/depletion behaviors. Figure 3a shows the schematic representation of the device setup: three skyrmion-conducting bars are connected by the junctions, and electric current is injected to the middle bars. We adopt the bridging junction of 200 nm in width and 10 μm in length (See scanning electron microscope (SEM) image in the right inset picture of Fig. 3a). On the basis of results in Fig. 2, the three skyrmion-conducting bars are considered to be well separated.

Figure 3b exemplifies the p-MOKE images under three different current densities as well as after the current is turned off. Here, 200 nm-width junctions are invisible due to the resolution limit of the MOKE microscopy. (See Methods.) Without the current injection on the middle bar, the spatial distribution of skyrmion bubbles is uniform, and the top, the middle, and the bottom bars are filled by the skyrmion bubbles with almost the same populations. By applying the current on the middle bar, the skyrmion bubbles are gathering at the upper edge similarly to the observations in Fig. 2. Important observation here is the difference in the skyrmion populations between the top and the bottom bars, even though the electric current is applied only on the middle bar. Note that the electric current does not flow along the junctions. As discussed below, we find the experimental evidence that the skyrmion pumping by current-exciting only the middle bar extends beyond several tens of micrometers, causing the imbalance in the skyrmion populations between the top and the bottom bars.

Figure 3c shows the numbers of skyrmion bubbles in the top bar *N*_top_, the bottom bar *N*_bottom_, and their sum *N*_top_ + *N*_bottom_ as the functions of the current density *j* applied on the middle bar. Note again that the current is applied only on the middle bar. With increasing *j*, *N*_top_ increases and *N*_bottom_ decreases and those saturates at around *j* ≈ 8.0~9.0 × 10^10^ A/m^2^. We find that *N*_top_ + *N*_bottom_ is almost kept constant, independent of *j*. We confirm that after the current on the middle bar is turned off, the skyrmion accumulation on the top bar and the depletion on the bottom bar are *relaxed* within several seconds, namely, the difference between *N*_top_ and *N*_bottom_ disappears and whole devise shows almost the same skyrmion spatial distribution as seen in the original one for *j* = 0 (see the right bottom panel of Fig. 3b and Supplemental Information 3). In addition to this, we do not see any hysteresis behavior for the current-induced change in *N*_top_ and *N*_bottom_.

There are some possibilities for the change in the number of skyrmion bubbles by the current injection, e.g., (i) local Joule heating effect, (ii) local Oersted field effect, and (iii) current-driven local skyrmion creation/annihilation. For (i): the middle bar is heated by the current. However, this heating effect cannot explain the asymmetric difference between *N*_top_ and *N*_bottom_. For (ii): the Oersted field induced by the current on the middle bar causes the difference between the magnetic field acting on the top and the bottom bars. However, the top (bottom) bar is spatially separated by 10 μm distance from the middle bar. As discussed in the measurements summarized in Fig. 2, the difference between *N*_top_ and *N*_bottom_ shown in Fig. 3c is too large to explain this by the local Oersted field effect. For (iii): current-induced skyrmion creation/annihilation in the system with spatially constricted geometry has been discussed in previous studies^22,49,50^. In the present experiment, a possible local spatial pattern constriction can occur at the junction points between the current-injected middle bar and the upper/lower bar. In this device (Fig. 3a,b), however, the junction width *w* = 200 nm, which is much smaller than skyrmion bubble size *d* (630 nm ⩽ *d* ⩽ 1040 nm), is too narrow to cause the current-induced local skyrmion creation/annihilation. In addition, no electric current is passing through the junction. Furthermore, the possibilities (i)∼(iii) discussed here can hardly explain why the summation of *N*_top_ and *N*_bottom_ is conserved.

We propose here the alternative and most plausible scenario to explain the observed features in Fig. 3; the skyrmion accumulation on the top bar, the skyrmion depletion on the bottom bar and the conservation of *N*_top_ + *N*_bottom_, can be interpreted in terms of the pumping of the skyrmion bubbles from the bottom bar to the top bar due to the skyrmion Hall effect occurring on the middle bar. By the current-injection on the middle bar, due to the skyrmion Hall effect, a motive force for the skyrmion bubbles perpendicular to the current direction occurs. Consequently, the middle bar pumps the skyrmion bubbles from the bottom bar to the top bar to reach the steady state with different skyrmion populations between these two bars.

Up to this stage, as shown in Figs. 2 and 3, we have experimentally examined the skyrmion accumulation and depletion via the sufficiently narrow gate channel(s) by the skyrmion Hall effect. Next, we show the experimental evidence that the skyrmion chemical potential consideration is essential for the nonlocal skyrmion accumulation and depletion phenomena in the length scale beyond several tens of micrometers.

### Skyrmion chemical potential

Figure 4a shows the schematic representation of the device setup: five skyrmion-conducting bars are connected by the junctions, and electric currents *j*_1_ and *j*_2_ are injected to the two separated bars (the gray regions). Those gray regions are connected to the top, the center and the bottom skyrmion bars by very narrow junctions (the hatched regions), where no electric current flow is anticipated. For the junction parts, we take 200 nm width and 8 μm length here, so that all the skyrmion bars are well separated. (See also the right inset of Fig. 3a and Supplementary Information 2.) As discussed above, the gray bars are anticipated to show the skyrmion pumping effect perpendicular to the current direction due to the skyrmion Hall effect; in the following *j*_1_ (*j*_2_) denotes the current density in the upper (lower) gray bar 1 (2) including its sign.

Figure 4b shows the p-MOKE images of the skyrmion device in the nonequilibrium steady states. Note again that the width of junctions is below the resolution limit, as well as the images in Fig. 3b. Here, we apply the currents with a density | *j*_1_ | = | *j*_2_ | = 5.0 × 10^10^ A/m^2^ on the skyrmion pumping bars 1 and 2. We observe the change in the skyrmion spatial distribution by the sign change of the currents *j*_1_ and *j*_2_. There are four cases. Case (I): (+, +), *j*_1_ and *j*_2_ are both positive. Case (II): (−, −), *j*_1_ and *j*_2_ are both negative. Case (III): (−, +), *j*_1_ is negative and *j*_2_ is positive. Case (IV): (+, −), *j*_1_ is positive and *j*_2_ is negative. For the definition of the sign of the currents *j*_1_ and *j*_2_, see also the current directions indicated by blue (+) and red (−) arrows in Fig. 4b.

The upper panel of Fig. 4c shows the numbers of skyrmion bubbles on the top (*N*_top_), the center (*N*_center_) and the bottom (*N*_bottom_) bars in the cases (I)~(IV). In Case (I), we see the clear difference in the skyrmion population among the top, the center and the bottom bars, i.e., the top (bottom) bar has largest (smallest) population. In Case (II), the sequential order of the skyrmion population among the top, the center and the bottom bars is reversed. In Case (III) (Case (IV)), the largest (smallest) skyrmion population is seen at the center bar and smaller (larger) skyrmion population is seen comparably at the top and the bottom bars. The lower panel of Fig. 4c shows the total number of skyrmion bubbles *N*_top_ + *N*_center_ + *N*_bottom_ for the cases (I)~(IV). We confirmed that *N*_top_ + *N*_center_ + *N*_bottom_ ∼ 3 × *N*_0_ in all the cases (I)~(IV), where *N*_0_ denotes the value in the equilibrium state without the current excitation, i.e., *j*_1_ = *j*_2_ = 0.

Let us first focus our attention on the result of Case (I). In this case, the bar 2 pumps an amount of skyrmion bubbles *N*_pump_ from the bottom bar to the center bar. Suppose that *N*_pump_ is determined by the current density. Because of *j*_1_ = *j*_2_ in Case (I), the bar 1 pumps the same amount of the skyrmion bubbles *N*_pump_ from the center bar to the top bar. This explains why the number of skyrmion bubbles on the center bar *N*_center_ is almost the same as *N*_0_ in the equilibrium system, as indicated by the horizontal gray bar in Fig. 4c. This amount of pumped skyrmion bubble *N*_pump_ is to be estimated by the difference in the numbers of skyrmion bubbles between the top and the center bars. At the same time, it should be the same as the difference in the numbers of the skyrmion bubbles between the center and the bottom bars. At least at this stage, the chemical potential for the skyrmion bubbles is not necessarily taken into account to explain the observed phenomena. However, the concept of the skyrmion chemical potential is indispensable to understand the result for Case (III): In Case (III), because the current *j*_1_ is reversed in comparison to Case (I), the increased (decreased) amount of the skyrmion bubbles on the center (top) bar is expected to be 2 × *N*_pump_ (1 × *N*_pump_) compared to *N*_0_ in the equilibrium system. However, the experimental results in the upper panel of Fig. 4c clearly show that the number of skyrmion bubbles on the center (top) bar in Case (III) is *smaller* (*larger*) than that on the top (bottom) bar in Case (I). This apparent discrepancy is to be solved by the following consideration for the skyrmion chemical potential.

Under the assumption that the net skyrmion bubbles can be described with a thermodynamic equation, the Helmholtz free energy F(*V*, *T*, *N*) of this closed system will be introduced using three thermodynamics quantities, volume *V*, temperature *T*, and the number of the skyrmion bubbles *N*. The total volume of three bars are fixed, and they are located on the common Si substrate which works as a heat bath of temperature *T*. Such setup leads to the conditions as *V* = const. and *T* = const. during pumping processes. Therefore, the skyrmion “*chemical potential*” *μ*(*T*, *V*, *N*) = −(*∂F*/*∂N*)_*T,V*_ will be the main quantity to decide the distribution of skyrmion bubbles at each bar.

Let us discuss the experimental results within the first order expansion of *μ* as a function of *N*. We use the notations *E*_*H*,1_ and *E*_*H*,2_ for the skyrmion motive forces by the skyrmion Hall effect on the skyrmion pumping bars 1 and 2, respectively. In the steady state, to compensate these *E*_*H*,1_ and *E*_*H*,2_, we introduce the relation as,

1 $$\left\{\begin{array}{c}{E}_{H,1}={\mu }_{{\rm{center}}}-{\mu }_{{\rm{top}}}=\frac{\partial \mu }{\partial N}({N}_{{\rm{center}}}-{N}_{{\rm{top}}})\\ {E}_{H,2}={\mu }_{{\rm{center}}}-{\mu }_{{\rm{bottom}}}=\frac{\partial \mu }{\partial N}({N}_{{\rm{center}}}-{N}_{{\rm{bottom}}})\end{array}\right.$$

for Case (III), where *μ*_top_, *μ*_center_ and *μ*_bottom_ are the chemical potentials of the skyrmion bubbles for the top, the center and the bottom bars, respectively. For Case (I), the relation should be,

2 $$\left\{\begin{array}{c}{E}_{H,1}={\mu }_{{\rm{top}}}-{\mu }_{{\rm{center}}}=\frac{\partial \mu }{\partial N}({N}_{{\rm{top}}}-{N}_{{\rm{center}}}),\\ {E}_{H,2}={\mu }_{{\rm{center}}}-{\mu }_{{\rm{bottom}}}=\frac{\partial \mu }{\partial N}({N}_{{\rm{center}}}-{N}_{{\rm{bottom}}}).\end{array}\right.$$

This consideration results in |*N*_top_ − *N*_center_| = |*N*_center_ − *N*_bottom_| = Δ*N* under the condition, | *j*_1_ | = | *j*_2_ |, i.e., |*E*_*H*,1_| = |*E*_*H*,2_|.

With these, under the condition that the sum *N*_top_ + *N*_center_ + *N*_bottom_ is conserved, the solutions of Eqs. (1) and (2) are,

3 $$\left\{\begin{array}{c}{N}_{{\rm{top}}}={N}_{0}+\varDelta N\\ {N}_{{\rm{center}}}={N}_{0}\\ {N}_{{\rm{bottom}}}={N}_{0}-\varDelta N\end{array}\right.\,{\rm{for}}\,{\rm{Case}}\,({\rm{I}}),$$

and

4 $$\left\{\begin{array}{c}{N}_{{\rm{top}}}={N}_{0}-\frac{1}{3}\varDelta N\\ {N}_{{\rm{center}}}={N}_{0}+\frac{2}{3}\varDelta N\\ {N}_{{\rm{bottom}}}={N}_{0}-\frac{1}{3}\varDelta N\end{array}\right.\,{\rm{for}}\,{\rm{Case}}\,({\rm{III}}),$$

where *N*_0_ is the number of skyrmion bubble in the system without current application, i.e., *j*_1_ = *j*_2_ = 0 system.

In the same way, for case (II) and Case (IV), the chemical potential consideration for the skyrmion bubbles gives,

5 $$\left\{\begin{array}{c}{N}_{{\rm{top}}}={N}_{0}-\varDelta N\\ {N}_{{\rm{center}}}={N}_{0}\\ {N}_{{\rm{bottom}}}={N}_{0}+\varDelta N\end{array}\right.\,{\rm{for}}\,{\rm{Case}}\,({\rm{II}}),$$

and

6 $$\left\{\begin{array}{c}{N}_{{\rm{top}}}={N}_{0}+\frac{1}{3}\varDelta N\\ {N}_{{\rm{center}}}={N}_{0}-\frac{2}{3}\varDelta N\\ {N}_{{\rm{bottom}}}={N}_{0}+\frac{1}{3}\varDelta N\end{array}\right.\,{\rm{for}}\,{\rm{Case}}\,({\rm{IV}}).$$

The calculated results in Eqs. (3–6) consistently agree with our experimental results for all cases. Thus, we conclude that the consideration of the skyrmion chemical potential explains the skyrmion pumping effect.

## Discussions

In the present study, we proposed the thermodynamic concept for the control of the skyrmion aggregate. We experimentally demonstrated that the nonlocal skyrmion accumulation/depletion can occur over the length scale beyond several tens of micrometers in Pt/Co/Ir heterostructure via the skyrmion Hall effect induced by the locally injected electric current. To describe the macroscopic response of the aggregate of the skyrmion bubbles to the current injection, we introduced the skyrmion chemical potential. For the verification of the new concept, we designed and developed the skyrmion devices including sufficiently narrow junction(s). We successfully controlled the skyrmion aggregate and realized the nonlocal skyrmion accumulation/depletion beyond several tens of micrometers, all of which can be well interpreted in terms of the skrymion chemical potential.

In the devices used in the present study, the skyrmion-conducting bars are connected by narrow gate channels which are always open. The gate function, i.e., its open/close operation, can be controlled by external stimuli such as local magnetic field, local electric field, local heating and so on. By the programming of the open/close of the skyrmion gate, the local accumulation/depletion at the aimed skyrmion bar will be maintained even after the current driven Hall effect is turned off.

In addition, details of the ultra-fast dynamics prior to achieve the nonequilibrium steady state will be interesting topics for further investigations, relating to complexities of skyrmion dynamics, such like plastic deformations and/or nucleation/annihilation during pumping processes.

The concept of skyrmion chemical potential can provide a fundamental insight into the skyrmion aggregative behavior as well as a new methodology for the skyrmion physics.

## Methods

### Sample preparation

The Ta(10 Å)/Pt(50 Å)/Co(*t*)/Ir(8 Å)/Pt(50 Å) multilayer was grown onto a Si substrate with 100-nm-thick thermally formed SiO_2_ layer by means of combining dc and rf magnetron sputtering techniques. We adopted rf sputtering only for Pt deposition so as to suppress net perpendicular magnetic anisotropy of the multilayer. The Co layer thickness *t* was continuously varied from 6*Å* ⩽ *t* ⩽ 7*Å* using a moving shutter function to meet the severe condition for room-temperature skyrmion bubbles in a single Pt/Co/Ir trilayer stack. Devices were patterned by means of standard photolithography, subsequent Ar ion milling and electron beam lithography.

### Film characterization

The film characterizations and their evaluation methods were equivalent to those of our earlier works in ref. ^47^. The saturation magnetization *M*_*s*_(=(3.0 ± 0.3) × 10^5^ A/m) and the effective perpendicular magnetic anisotropy *K*_*u*_(=(9.2 ± 0.4) × 10^4^ J/m^3^) were obtained by vibrating sample magnetometry. The Dzyaloshinskii-Moriya constant *D* was estimated from the periodicity of stripe domains using a MOKE imaging, as |*D*| = 1.1 ± 0.2 mJ/m^2^. (See also Supplementary Information 1).

### Skyrmion counting system

The skyrmion bubbles settled down the nonequilibrium steady state within a few second, and such nonequilibrium states were observed substantially wide electric current density *j* region of 3 × 10^10^ A/m^2^ < *j* < 2 × 10^11^ A/m^2^. Differential polar magneto-optical Kerr effect imaging experiments were performed at room temperature using a commercial MOKE microscope from Evico Magnetics with a spatial resolution of 250 nm/pixel. The p-MOKE data were captured as 72 dpi bitmap images, and then converted into binary data by setting the appropriate threshold value. The number of skyrmion bubbles, sizes, and their standard deviations were analyzed by using the particle counting method. Smaller particle less than 0.3 μm^2^ were excluded as noises.

Since the differential imaging processing was adopted, an image drift sometimes caused enhanced contrast in edge regions. This affected the visibility of the 200 nm width junction(s) in Figs. 2–4, where the larger drift resulted in appearance of the junction only in Fig. 2b.

## Supplementary information

Supplementary Information

## Data Availability

The data that support the plots within this paper and other findings of this study are available from the corresponding author upon reasonable request.
