# Supplementary material for: Nonlocal accumulation, chemical potential, and Hall effect of skyrmions in Pt/Co/Ir heterostructure
Source: Sci Rep. 2020 Jan 23;10:1009. doi: 10.1038/s41598-020-57818-w (PMC6978358; doi:10.1038/s41598-020-57818-w)
Supplement: Supplementary file 1 — Supplementary Information [file 41598_2020_57818_MOESM1_ESM.pdf]

Supplementary Information to "Nonlocal accumulation, chemical potential, and Hall effect of skyrmions in Pt/Co/Ir heterostructure"

S. Sugimoto,[1] W. Koshibae[2], S. Kasai,[1,3,*] N. Ogawa,[2,3] Y. K. Takahashi,[1] N. Nagaosa,[2,4] and Y. Tokura[2,4,5]

[1]*Research Center for Magnetic and Spintronic Materials, National Institute for Materials Science (NIMS), 1-2-1 Sengen, Tsukuba 305-0047, Japan*
[2]*RIKEN Center for Emergent Matter Science (CEMS), Wako 351-0198, Japan*
[3]*JST, PRESTO, 4 - 1 - 8 Honcho, Kawaguchi, Saitama 332 - 0012, Japan*
[4]*Department of Applied Physics, University of Tokyo, Tokyo 113-8656, Japan*
[5]*Tokyo College, University of Tokyo, Tokyo 113-8656, Japan*

1. Domain morphologies of skyrmion bubbles in Pt/Co/Ir trilayers

The field dependence of magnetic domains of a single Pt/Co/Ir trilayer stack is presented in Fig. S1a as the subsequent p-MOKE images. The images are clipped from a field sweep from negative saturation to positive saturation $(-10 \leq H \leq +10\ \text{Oe})$. Starting from uniform magnetization, tiny circular positive magnetization domains (skyrmion bubbles with their core polarizations are positive, i.e., *core-up skyrmion bubbles*) nucleate and subsequently increase in number density as the field increases (see the case at $H = -3.2\ \text{Oe}$). Around $H = -1.5\ \text{Oe}$, some of these core-up skyrmion bubbles elongate into a stripe shape that dominate the entire area of the film at $H = -0.1\ \text{Oe}$. Increasing the external field to the positive side, the gray positive magnetization area expands, and stripe domains start to be torn apart and turned into opposite skyrmion bubbles (*core-down skyrmion bubbles*), at $H = +1.0\ \text{Oe}$. These core-down skyrmion bubbles increase in number and replace all remaining stripe domains around $H = +3.4\ \text{Oe}$. The obtained skyrmion bubble phase does not show a complete periodicity in their arrangement like a skyrmion lattice, due to inhomogeneity in the ultrathin ferromagnetic layer. Applying a higher field ($H = +5.7\ \text{Oe}$) reduces the number of core-down skyrmions and slightly changes their individual size. All core-down skyrmion bubbles are annihilated around $H = +8.2\ \text{Oe}$. The observed skyrmion size, on a sub-μm scale, where the contribution of the dipolar field plays a dominant role [1].

Figure S1b shows the average diameters of the core-up and core-down skyrmion bubbles $\langle d \rangle$ as well as the widths of the stripe domains with varying H. The width of the stripe domain is determined by the balance of the demagnetizing energy and the domain wall surface energy including DMI as $\sigma_{\text{DW}} = 4\sqrt{AK_{\text{u}}} - \pi|D|$ [2,3]. The domain wall surface

energy density σ_{DW} was calculated from the periodicity of stripe domains $d = d_{\uparrow} + d_{\downarrow} = 1.97 \pm 0.35\ \mu\mathrm{m}$ for $H = -0.1\ \mathrm{Oe}$ as $\sigma_{\mathrm{DW}} = 1.6\ \mathrm{mJ/m^2}$, and hence $|D|$ can be estimated as $|D| = 1.1 \pm 0.2\ \mathrm{mJ/m^2}$ using $A = 1 \times 10^{-11}\ \mathrm{J/m}$. Obtained $|D|$ should be large enough for inducing Néel-type skyrmion bubbles. It should be noted that these domain morphologies are quite sensitive to the thickness variation ($\Delta t \ll 1\mathrm{Å}$). That is, therefore, we set tiny differences in the field amplitude as $H = +3.8\ \mathrm{Oe}$ for Figs. 2 and 3, and as $H = +2.7\ \mathrm{Oe}$ for Fig. 4 in the main text, so as to obtain comparable densities/sizes of skyrmion bubbles between different films.

2. Local Oersted field effect

Control experiments are performed to access proximity effects of local Oersted field induced by neighboring skyrmion-conducting bars. Two types of control samples are prepared with analogies of Fig. 2a and Fig, 4a in the main text, where designs of skyrmion-conducting bars and their separation distance(s) are identical, and only the junctions are removed.

The control experiment for Fig. 2a is introduced in Fig.2c in the main text: Results of imbalance in the numbers of skyrmion bubbles between the top bar N_{top} and the bottom bar N_{bottom} without the junction show a deviation from 1, as $N_{\mathrm{top}}/N_{\mathrm{bottom}} \sim 1.2$. On the other hand, the system with the junction shows $\overline{N_{\mathrm{top}}/N_{\mathrm{bottom}}} = 2.3 \pm 0.1$.

The control experiment for Fig. 4a is summarized in Fig. S2. The p-MOKE image of original (control) sample at the nonequilibrium steady states is shown in the upper (lower) panel of Fig. S1a. These setups are identical with Case (I) of Fig. 4b in the main text, i.e., the positive currents $5.0 \times 10^{10} \mathrm{A/m^2}$ are locally applied on the second and forth skyrmion-conducting bars as indicated blue arrows in Fig. S2a.

At the original sample in the top panel of Fig. S2a, we see the clear difference in the skyrmion populations: The top bar has the largest population, and the populations of the top, the center, and the bottom bars are decreasing in a sequential order, from top to bottom. At the control sample shown in the lower panel of Fig. S2a, we also observe small differences in the skyrmion populations between the top, the center, and the bottom bars, but these differences are much obscure compared with those in the original sample. The skyrmion population at the top bar of the control sample is much smaller than that of the original sample, and is almost comparable with the population of the middle bar.

Figure S2b shows the number of skyrmion bubbles N_i $(i = \mathrm{top, center, bottom})$ in the original and control samples. The data of the original sample are shown as the results for Case (I) in Fig.4c in the main text. We see that the differences in N_i in control sample are much smaller than those in the original sample.

3. Time evolution of skyrmion relaxation

Additional experiments are performed to access time evolutions of skyrmion relaxation process with three skyrmion-conducting bar setup used in Fig. 3 in the main text. The local current $8.0 \times 10^{10}\ \mathrm{A/m^2}$ is injected to the middle bar which triggers skyrmion accumulation (depletion) in the top (bottom) bar following manner of Fig. 3. Once skyrmion bubbles stay at the nonequilibrium steady states, the local current is turned off, and subsequent time evolution of domain texture is captured by p-MOKE imaging.

Figure S3 show the time evolution of the numbers of skyrmion bubbles in the top bar N_{top} and bottom bar N_{bottom} accompaning their ratio $N_{\mathrm{top}}/N_{\mathrm{bottom}}$. The shutoff of local current is set at $t = 0$ here. The clear imbalance in the skyrmion populations between the top and the bottom bars as $N_{\mathrm{top}}/N_{\mathrm{bottom}} = 1.5 \pm 0.1$ at $t = 0$ indicates skyrmion accumulation and depletion are induced in the same manner with Fig. 3. Once the local current is turned off, the imbalance starts to decay, and decreases to $N_{\mathrm{top}}/N_{\mathrm{bottom}} \sim 1$ around $t = 5$ s. The exponential decay function, black solid line in the bottom panel, nicely follows experimental results with estimated decay constant $\tau = 3.7 \pm 1.2$ s.

Focusing on microscopic origins of skyrmion populations, their time scale is considered to depend on (i) single skyrmion dynamics including nucleation/annihilation and deformation processes (nanosecond scale) [4,5], (ii) commensuration effects proposed in disordered magnets (calculated as micro-second scale) [6,7], or (ii) thermal diffusion processes (milli-second to second scale in metallic films). Since our p-MOKE imaging technique does not support any time-resolved setup, we will never access fast transient evolution within scope of this paper. However, above relaxation process appears as much slower dynamics characterized by several seconds scale in decay constant τ. These results imply the contributions of the thermal diffusion process are not negligible there.

[1] Montoya, S. A., Couture, S., Chess, J. J., Lee, J. C. T., Kent, N., Henze, D., Sinha, S. K., Im, M. Y., Kevan, S. D., Fischer, P., McMorran, B. J., Lomakin, V., Roy, S. & Fullerton, E. E.
Tailoring magnetic energies to form dipole skyrmions and skyrmion lattices.
Phys. Rev. B **95**, 024415 (2017).
[2] Heide, M., Bihlmayer, G. & Blügel, S.
Dzyaloshinskii-Moriya interaction accounting for the orientation of magnetic domains in ultrathin films: Fe/W(110).
Phys. Rev. B **78**, 140403 (2008).
[3] Thiaville, A., Rohart, S. Jué, E., Cros. V. & Fert, A.
Dynamics of Dzyaloshinskii domain walls in ultrathin magnetic films.
Europhys. Lett. **100**, 57002 (2012).
[4] Onose, Y., Okamura, Y., Seki, S., Ishiwata, S. & Tokura, Y.
Observation of Magnetic Excitations of Skyrmion Crystal in a Helimagnetic Insulator Cu_2OSeO_3.
Phys. Rev. Lett. **109**, 037603 (2012).
[5] Büttner, F., Lemesh, I., Schneider, M., Pfau, B., Günther, C. M., Hessing, P., Geilhufe, J., Caretta, L., Engel, D., Krüger, B., Viefhaus, J., Eisebitt, S. & Beach, G. S. D.
Field-free deterministic ultrafast creation of magnetic skyrmions by spin–orbit torques.
Nature Nanotech. **12**, 1040-1044 (2017).
[6] Koshibae, W. & Nagaosa, N.
Theory of current-driven skyrmions in disordered magnets.
Sci. Rep. **8**, 6328 (2018).
[7] Reichhardt, C., Ray, D. & Reichhardt, C. J. O.
Nonequilibrium phases and segregation for skyrmions on periodic pinning arrays.
Phys. Rev. B **98**, 134418 (2018).

Supplemental Figure S1. Field evolution of skyrmion bubbles/stripe domains morphologies.

(a) The p-MOKE images of Field evolution of skyrmion bubbles/stripe domains morphologies. All images are firstly normalized by the ones taken at negative saturation field $H = 10.0\ \mathrm{Oe}$. Red and blue enclosed images correspond to core-up and core-down skyrmion bubbles, respectively. (b) Field dependence of the averaged size of each spin texture: the diameter of core-up skyrmion bubbles plotted by red symbols, the narrower width of stripe domains by open symbols, and the diameter of core-down skyrmion bubbles by blue symbols.

Supplemental Figure S2. Skyrmion distributions of closely allocated wires with or without bridge junctions.

(a) The p-MOKE images of the original sample (top panel: with junctions) and the control sample (bottom panel: without junctions) observed under the condition with $j_1 = j_2 = +5.0 \times 10^{10}\ \mathrm{A/m^2}$ and $H = +2.7\ \mathrm{Oe}$. (b) The number of skyrmion bubbles at the top, center, and bottom bars with and without junctions. The closed circles indicate results of the original sample, and open circles do those of the control sample. The sample configurations, current amplitude, and field condition are identical with those of Case (I): $(+,+)$ in Fig. 4b in the main text.

Supplemental Figure S3. Time evolution of imbalanced skyrmion populations.

Time evolution of the numbers of skyrmion bubbles in the top bar N_{top}, the bottom bar N_{bottom} (top panel), and their ratio $N_{\mathrm{top}}/N_{\mathrm{bottom}}$ (bottom panel). The device setup is common with three skyrmion-conducting bars in Fig. 3 in the main text, under the condition with $j = 8.0 \times 10^{10}\ \mathrm{A/m^2}$ and $H = +4.1\ \mathrm{Oe}$. N_{top}, N_{bottom}, and $N_{\mathrm{top}}/N_{\mathrm{bottom}}$ are plotted by red, blue, and black circles respectively. The fitting results by the exponential decay curve is superimposed by the black solid line in the bottom panel.

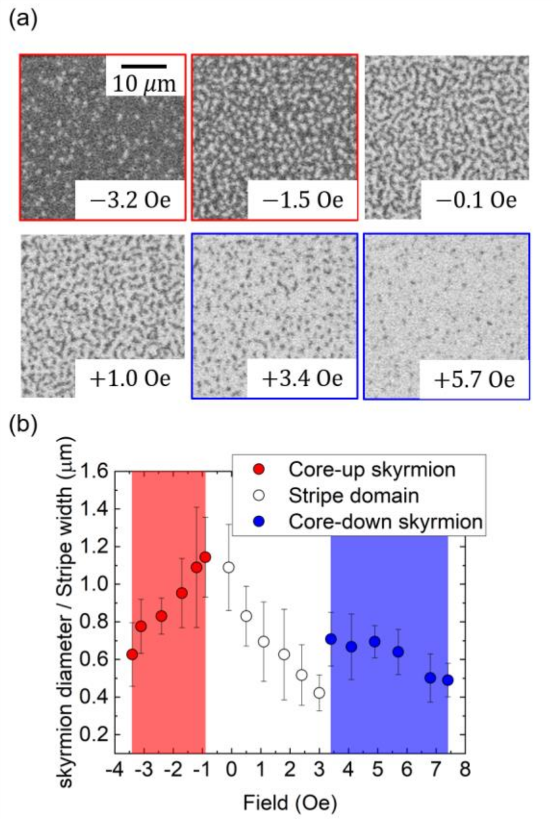

Supplementary Figure S1

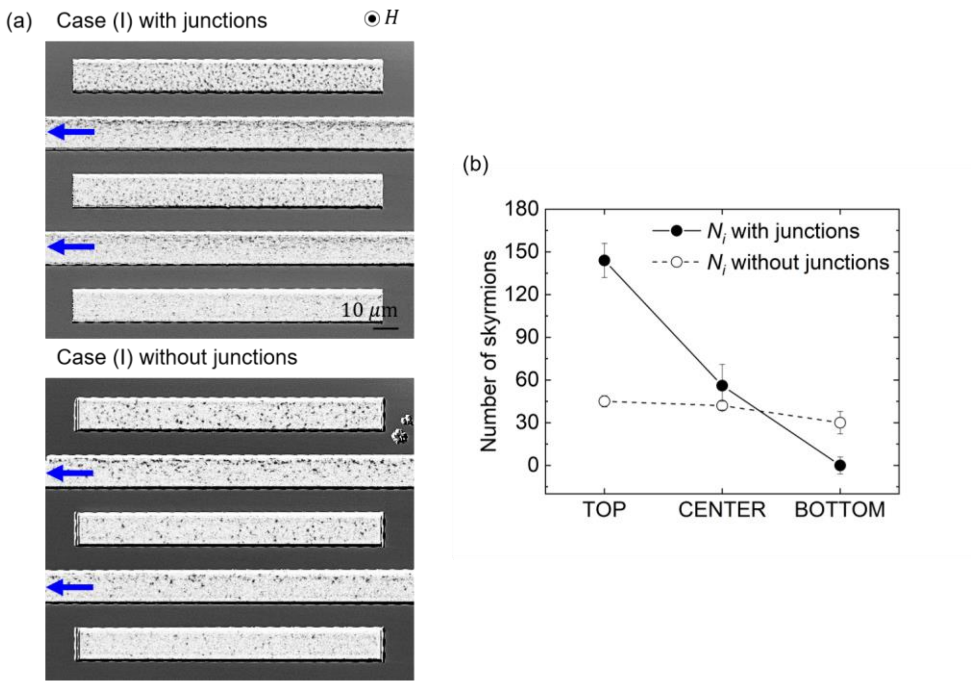

Supplementary Figure S2

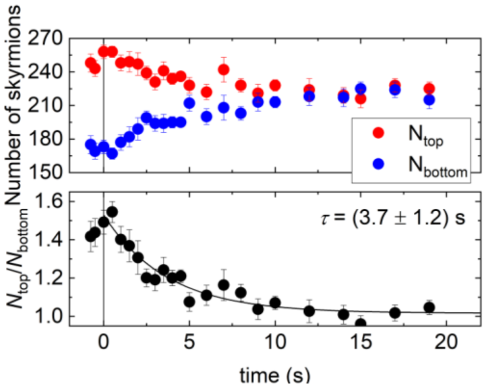

Supplementary Figure S3
